# The impact of physical exercise, health effect, and harmony effect on young adults’ fertility attitudes: a study based on empirical analysis of 2023 CGSS data

**DOI:** 10.3389/fpubh.2025.1556559

**Published:** 2025-11-13

**Authors:** Xiuxiu Bu, Linbo Lu, Xinze Li, Cong Cen

**Affiliations:** 1 College of Physical Education, Hunan Normal University, Changsha, Hunan, China; 2 College of Physical Education, Qiannan Normal University for Nationalities, Duyun, Guizhou, China; 3 School of Humanities, Beijing Sport University, Beijing, China; 4 College of Physical and Health Education, East China Jiaotong University, Nanchang, Jiangxi, China

**Keywords:** physical exercise, fertility attitudes, health effect, harmony effect, Chinese General Social Survey

## Abstract

**Introduction:**

Faced with a declining birth rate, China urgently needs to identify effective strategies to improve residents’ attitudes toward having additional children. This study investigates whether physical exercise, grounded in leisure theory and demographic principles, serves as a significant factor in promoting positive fertility attitudes.

**Methods:**

This research employs a conceptual framework that identifies the prerequisites and inherent benefits of physical exercise. We then empirically test its influence using data from the 2023 Chinese General Social Survey (CGSS). The desire for another child is the key outcome variable, regressed on measures of sports participation. Robustness checks are conducted to address potential endogeneity, and a mediation analysis is performed to uncover the underlying pathways.

**Results:**

The results demonstrate that participation in sports activities substantially improves individuals’ fertility attitudes. This positive effect remains robust after controlling for endogeneity. The mediation analysis reveals that the relationship is driven by two significant pathways: first, through the enhancement of physical and mental health, and second, through the strengthening of family cohesion and overall life satisfaction.

**Discussion:**

The findings confirm that physical exercise is a significant and positive predictor of fertility attitudes in China. The mechanisms operate through both bio-psychological and socio-familial channels. We conclude that public policies aimed at promoting accessible and appealing physical exercise can effectively complement existing fertility support measures, thereby providing a viable strategy to help optimize China’s national population policies.

## 1 Introduction

Since the early 21st century, fertility attitudes have reemerged as a central lens for understanding fertility behavior. In China, this renewed attention reflects not only the forward-looking nature of fertility attitudes but also mounting demographic pressures—persistently low fertility, population aging, and sex imbalance. Although the state sequentially introduced the “selective two-child” (2013), “universal two-child” (2015), and “universal three-child” (2021) policies, these measures did not yield the anticipated rebound in births. A common interpretation is that rapid economic development and aspirations for upward mobility crowd out the desire for childbearing (1). Yet this framing is incomplete: if “fewer children” maximizes mobility, why do fertility rates not collapse to zero? Even in contexts of ultra-low fertility, births persist at non-trivial levels, implying that non-economic forces sustain fertility intentions and behavior (2).

Against this backdrop, contemporary Chinese youth face fast-paced, competitive social environments and adopt physical exercise as a salient coping and self-development practice. Beyond improving health and relieving stress, physical exercise can foster self-efficacy, social bonding, and life satisfaction—factors plausibly linked to family formation preferences. As productivity rises and living standards improve, demand for physical exercise has grown, suggesting that lifestyle factors may intersect with fertility attitudes in policy-relevant ways. If physical exercise shapes the psychosocial foundations of fertility decision-making, then aligning fertility support with sports participation could amplify policy effectiveness and promote a more balanced population structure.

Considering that young people are the key driving force of social vitality and development, their fertility status reflects the overall fertility capacity of society, and changes in their fertility attitudes have a decisive impact on national fertility rates. The findings of this study have clear practical applications. Practically, this perspective offers levers for organizations and governments: employers can support physical exercise to enhance well-being and cohesion among reproductive-age workers; digital platforms can help identify sports preferences and participation intensity to better target fertility support; and investment in experiential segments of the sports industry can complement rural revitalization by strengthening community life and family orientation.

Prior studies, gap, and this paper’s contribution. Existing research on fertility attitudes in China has emphasized economic constraints (e.g., costs of children, career–family trade-offs) with comparatively less attention to lifestyle and health behaviors; where such factors are considered, evidence is often descriptive, cross-sectional, or extrapolated from non-Chinese settings, and mechanisms remain under-specified. This paper addresses that gap by investigating the relationship between physical exercise and fertility attitudes among contemporary Chinese youth, articulating testable psychosocial mechanisms, and providing nationally representative evidence with robustness checks and heterogeneity analyses. In doing so, it reframes fertility attitudes through a health effect or harmony effect lens and offers policy-relevant pathways that complement economic interventions.

## 2 Literature review and research hypothesis

Physical exercise is a distinctive type of consumption behavior, influenced by both external conditions and internal psychological factors. External conditions include material resources and leisure time, while internal psychological factors refer to leisure needs. Fundamentally, physical exercise involves how individuals perceive the role of consumption in their lives, particularly the relationship between enjoyment and development needs. From this perspective, there exists an intrinsic link between physical exercise and fertility attitudes. Young people’s fertility attitudes are influenced by a complex interplay of economic and non-economic factors. Economic factors include socioeconomic status (19), population mobility, social security, and digital lifestyles. Non-economic factors span various dimensions at both individual and family levels. Research indicates that higher education levels, good physical and mental health, greater happiness, and strong marital relationships significantly enhance fertility attitudes among young people (20).

From the health perspective, physical exercise can improve physical health. While individuals choose different leisure activities based on their values and needs, all forms of physical exercise can positively impact the health of young people who are under significant stress. According to the World Health Organization (WHO), health includes not only physiological well-being but also mental health and social adaptation. Appropriate leisure and exercise during leisure time have a positive effect on an individual’s physical, psychological, social, and environmental interactions, thereby improving their overall quality of life. Health is universally acknowledged as an important factor influencing fertility attitudes (3). If an individual’s physical and mental health is not well, it can suppress their fertility attitudes. This manifests in two main ways: first, health directly affects fertility capacity and the quality of life, especially for women (4); second, health is the premise for optimal reproduction, as unhealthy parents increase health risks for their children and raise the care costs for children, further reducing fertility attitudes (5). Therefore, this paper posits that physical exercise may influence fertility attitudes through health effects.

From the perspective of happiness, physical exercise can enhance both individual and family happiness. The emotional foundation of happiness comes from harmonious relationships at both the family and societal levels: (1) At the family level, residents’ happiness comes from harmonious work and family relationships. Studies by Izzo, F. et al. suggest that happiness exists as a positive spillover effect from family connections, where a better family atmosphere improve individual happiness (6). Mengmeng Zhou et al. indicated that “work-family” conflicts have a significant impact on job burnout, performance, and subjective well-being (7). Zabriskie & McCormick pointed out that a “balanced family leisure model” better promotes the adaptability of family functions, thereby enhancing individual and family happiness (8). Supporting this view, Ewa Jarosz highlights that sports activities help alleviate work fatigue and address existing health issues among women, while also offering enjoyment and fulfilling personal goals or lifestyle preferences, ultimately enhancing women’s fertility attitudes (Jarosz et al., 2023). (2) At the societal level, residents’ happiness comes from the harmony and stability of the social environment. Helliwell & Putnam suggested that social trust is the emotional foundation of modern happiness. Mao Xiaoping highlighted that social disarray reduces happiness levels (9). Sports have a unique social function, improving the social trust environment. Physical exercise has become a new form of social interaction among the younger generation, to some extent bridging the trust gap between people. Therefore children of migrant families who actively participate in sports positively influence the quality of “sports friendships” with urban peers, which, in turn, positively affects the level of trust toward urban peers.

From the perspective of happiness, scholars generally agree that physical exercise enables individuals to exercise free choice, enhance their sense of competence and belonging, and improve intrinsic motivation, thereby strengthening family happiness. This underscores the close connection between sports activities and daily life, making them an effective means of improving quality of life and family well-being. Supporting this view, Ruseski et al. found that individuals who regularly participate in sports activities tend to exhibit higher levels of happiness, as physical exercise enhances social capital, which in turn boosts personal family satisfaction (10). Leibenstein further posits that the benefits of having children are primarily reflected in intangible intrinsic happiness, while the costs can be assessed from an economic perspective. If individuals perceive that the family happiness gained from having children outweighs the economic costs, they are more inclined to choose parenthood (11). Social psychology theories suggest that personal emotional experiences and life perceptions significantly influence young people’s fertility attitudes. Icek Ajzen’s research further highlights that positive attitudes toward fertility and the expectation of deriving happiness from having children significantly strengthen fertility attitudes, potentially leading to higher actual fertility rates (12). Therefore, this paper suggests that physical exercise, through its impact on trust and happiness, positively influences fertility attitudes.

Additionally, from the perspective of internet usage, physical exercise reduces indoor time, thus reducing internet usage and online consumption behaviors to some extent. This improve communication among family members and improves family harmony. Related studies indicate that internet use can suppress fertility attitudes, mainly by exposing individuals to negative information, changing fertility perceptions, and increasing the costs of child-rearing (13).

However, physical exercise is an economic behavior requiring individuals to invest both time and money. Through sports, individuals can gain subjective benefits, which are often positive. Similarly, fertility decisions involve a cost–benefit analysis, where individuals weigh the direct and indirect costs of having another child, typically considering that additional children bring future positive returns to the family. Materially, physical exercise can increase the cost of child-rearing. The sports economy is becoming a new consumer trend, with consumers frequently increasing their expenditures once they become engaged in sports activities (14). In terms of time, physical exercise can conflict with child-rearing. According to Zhiwei Li, the reduced leisure time resulting from child-rearing improve indirect costs, further weakening women’s fertility attitudes. Those with a negative view of leisure often consider physical exercise as a lifestyle for the wealthy and leisure-oriented individuals, typically high-income groups (15). High income does not necessarily lead to higher fertility attitudes. Kearney and Levine points out that high-income groups’ consumption behaviors, particularly status consumption, may be a significant cause of low fertility behavior (16). In light of these perspectives, physical exercise may exert a negative influence on fertility attitudes due to the additional time costs involved and constraints arising from limited financial resources. Based on this, the paper proposes the following hypotheses:

*H1*: Physical exercise has a positive impact on fertility attitudes among the reproductive-age population.

*H2*: Physical exercise positively impacts fertility attitudes through health effects.

*H3*: Physical exercise positively impacts fertility attitudes through harmony effects.

*H4*: Physical exercise may exert a negative influence on fertility attitudes owing to additional time costs and constraints imposed by limited financial resources.

## 3 Methodology and data

### 3.1 Data sources

This study utilizes data from the China General Social Survey (CGSS) 2023, which collects comprehensive information on the behaviors, attitudes, and socio-economic conditions of Chinese citizens, reflecting societal changes over time. To ensure data accuracy, the following data preprocessing steps were undertaken: (1) The study focuses on the reproductive-age population, with male participants aged 22–60 and female participants aged 20–49 (17). (2) Missing, extreme, and abnormal values were excluded. Consequently, 2039 valid samples were obtained.

### 3.2 Description of variables

#### 3.2.1 Explained variables

This study primarily builds upon the classic demographic theory of the “marginal child cost-utility theory,” considering physical exercise as a form of consumption behavior. It is hypothesized that sports activities may exert time and economic pressure on fertility costs, requiring reproductive-age couples to evaluate whether this demand conflicts with their intention to have one or more children. The aim is to emphasize that fertility should occur without compromising existing quality of life. Therefore, this study proposes using fertility attitudes as the dependent variable, replacing the traditional approach of measuring fertility attitudes using ideal family size. For transparency, we acknowledge that the gap measure may capture realized constraints in addition to preferences. Accordingly, we conduct robustness test using alternative outcomes.

Construction of the fertility attitudes Variable: The difference between the ideal number of children and the actual number of children is used to measure fertility attitudes. A positive difference (excluding zero) indicates a willingness to have more children, which is assigned a value of 1, while no intention is assigned a value of 0. Ideal Number of Children: Measured by the response to the CGSS 2023 question, “If there were no policy restrictions, how many children would you want?” Actual Number of Children: Measured by the response to the CGSS 2023 question, “How many children do you have (including stepchildren, adopted children, and children who have passed away)?”.

#### 3.2.2 Explanatory variables

According to the CGSS 2023 data, the frequency of sport participation activities over the past 12 months is measured using the question: “In the past 12 months, how often did you participate in sports activities” The five response options range from “Never” to “Almost every day,” with values assigned from 1 to 5.

#### 3.2.3 Control variables

To control for external factors affecting the analysis results and enhance the robustness of the model, this study refers to the research of Wesolowski (18), selects relevant control variables at the individual, family, and societal levels. The definitions of control variable are shown in Table 1.

**Table 1:** Control variable settings and descriptions.

Variable type	Variable name	Variable declaration
Individual	Gender	1 = male, 0 = female
	Age	2023 minus year of birth
	Education level	0 = no formal education; 1 = primary school; 2 = junior high; 3 = vocational high (general high/tech secondary); 4 = college diploma/bachelor’s; 5 = master’s degree or above
	Household registration	1 = non-agricultural household registration (formerly non-agricultural), 0 = otherwise
	Political affiliation	1 = Communist Party member, 0 = others
	Religious belief	0 = no religious belief, 1 = any religious belief
	Ethnicity	1 = Han, 0 = other ethnicities
	Marital status	1 = currently married or remarried(first marriage or remarriage); 0 = unmarried, divorced, or widowed
	Fertility history	1 = has had at least one biological child (including deceased); 0 = none
	Older support	1 = believes older adult should primarily bear responsibility for their own old-age support; 0 = otherwise
	Migration	1 = household registration has changed; 0 = otherwise
Family	household size	Number of other people living in the household
	Housing Ownership	1 = owns housing; 0 = otherwise
	Economic Status	1 = household per capita income above local per capita average; 0 = otherwise
Societal	Region	1 = eastern region, 2 = central region, 3 = western region
	Pension Insurance	1 = participates in urban/rural basic pension or new rural social pension; 0 = otherwise
	Medical Insurance	1 = participates in basic medical insurance or new rural cooperative/public medical insurance; 0 = otherwise

#### 3.2.4 Mediator variables

We treat health effect as the first mediator, proxied by three CGSS 2023 items: (i) self-rated overall health (1 = very unhealthy to 5 = very healthy); (ii) functional/physical health (frequency of activity limitation due to health problems in the past 4 weeks: 1 = always to 5 = never); and (iii) mental health (frequency of feeling depressed in the past 4 weeks: 1 = always to 5 = never).

Another mediating effect is the harmony effect. We measure interpersonal trust with the item “Generally speaking, most people can be trusted” (1 = strongly disagree; 5 = strongly agree) and subjective well-being with “Overall, how happy do you feel with your life?” (1 = very unhappy; 5 = very happy).

#### 3.2.5 Moderation variables

Financial and temporal constraints are operationalized from the CGSS 2023 questionnaire. A household is defined as Affluent (coded 1) if per-capita income exceeds the average of the respondent’s registered locality; otherwise it is Non-Affluent (coded 0). For time, respondents reporting more than 22 workdays in the past month are classified as Works Overtime (coded 1), others as 0.

### 3.3 Model construction

The dependent variable in this study is fertility attitudes, coded as a binary outcome. Accordingly, we employ a logistic regression model and the subgroup regressions model to test hypotheses 1–4. The model is specified as Equation 1:


$$F_{i}=\beta_{0}+\beta_{1}S_{i}+\beta_{p}\text{Contro}l_{p}+\epsilon_{1}$$ (1)

Where: $F_{i}$ is the fertility attitudes of individual; $S_{i}$ denotes the frequency of physical exercise activities of individual *i*; $\text{Contro}l_{p}$ is a vector of control variables; and $\epsilon_{1}$ is the error term.

## 4 Results

### 4.1 Descriptive statistics

The sample was partitioned into two groups based on respondents’ fertility attitudes. As shown in Table 2, 901 individuals reported a willingness to have additional children. Relative to those without such intentions, this group exhibits significantly higher educational attainment and per-capita household income, as well as a lower mean age, with all between-group differences reaching statistical significance. Furthermore, respondents with fertility attitudes report a higher frequency of physical exercise, providing preliminary support for hypothesis 1.

**Table 2:** Table of descriptive statistics (*N* = 2039).

Variable name	G1	Mean	Min	Max	G2	Mean	Min	Max
Fertility attitude	1,138	0.34	0	1	901	0.36	0	1
Physical exercise	1,138	2.482	1	5	901	2.7	1	5
Gender	1,138	0.48	0	1	901	0.555	0	1
Age	1,138	32.8	20	60	901	33.6	20	60
Education level	1,138	2.3	0	5	901	2.6	0	5
Household registration	1,138	0.56	0	1	901	0.58	0	1
Political affiliation	1,138	0.14	0	1	901	0.18	0	1
Religious belief	1,138	0.21	0	1	901	0.19	0	1
Ethnicity	1,138	0.93	0	1	901	0.95	0	1
Marital status	1,138	0.86	0	1	901	0.9	0	1
Fertility history	1,138	0.75	0	1	901	0.88	0	1
Elderly support	1,138	0.43	0	1	901	0.46	0	1
Migration	1,138	0.2	0	1	901	0.22	0	1
Household size	1,138	2.8	0	10	901	3.2	0	10
Housing ownership	1,138	0.78	0	1	901	0.8	0	1
Economic status	1,138	0.47	0	1	901	0.55	0	1
Region	1,138	1.89	1	3	901	1.95	1	3
Pension insurance	1,138	0.84	0	1	901	0.86	0	1
Medical insurance	1,138	0.95	0	1	901	0.96	0	1

### 4.2 Results of baseline regression, path analysis, and moderation analysis

#### 4.2.1 Baseline regression

The primary focus of this study is the impact of physical exercise on residents’ fertility attitudes. The regression results are reported in Table 3. Model M1 includes only control variables; Ethnicity (*β* = 0.289, *p* < 0.10), Region (*β* = −0.014, *p* < 0.05), Household Size (*β =* −0.190, *p <* 0.10), Parity (*β =* −0.399, *p* < 0.01), Marital status (*β =* −0.401, *p* < 0.01), Pension Insurance (*β* = 0.161, *p <* 0.10), Education Level (*β =* 0.096, *p <* 0.05), and Gender (*β* = 0.249, *p* < 0.01) show statistically significant effects. In Model M2, after adding Physical Exercise, its coefficient is 0.012 (SE = 0.005), indicating a positive association with Fertility Attitude.

**Table 3:** Regression results of physical exercise on young people’s fertility attitudes.

Variables	Fertility attitude		First-birth intention	Second-birth intention
	M1	M2	M3	M4
Physical exercise		0.012 (0.005)	0.153 **(0.074)	0.070 *(0.039)
Age	−0.003 (0.005)	−0.003 (0.005)	0.012 (0.010)	0.006 (0.005)
Household registration	0.058 (0.090)	0.066 (0.090)	0.487 ***(0.154)	0.261 ***(0.092)
Religious belief	0.283 (0.173)	0.276 (0.173)	0.203 (0.376)	0.112 (0.196)
Ethnicity	0.289 *(0.151)	0.307 *(0.151)	0.231 (0.296)	−0.514 ***(0.189)
Region	−0.014 **(0.008)	−0.014 **(0.008)	−0.037 **(0.015)	−0.009 (0.010)
Household size	−0.190 *(0.074)	−0.185 **(0.074)	−0.120 (0.140)	−0.542 **(0.190)
Home ownership	−0.001 (0.106)	−0.002 (0.106)	−0.005 (0.180)	−0.001(0.099)
Parity	−0.399 ***(0.086)	−0.406 ***(0.086)	−0.322 **(0.190)	−0.298 **(0.095)
Marital status	−0.401 ***(0.106)	−0.405 ***(0.106)	0.521 ***(0.178)	0.212 **(0.106)
Medical insurance	−0.024 (0.185)	0.209 (0.185)	0.618 **(0.245)	0.221 (0.184)
Pension insurance	0.161 *(0.093)	0.158 *(0.093)	0.062 (0.170)	0.063 (0.096)
Education level	0.096 **(0.043)	0.091 *(0.043)	−0.118 (0.085)	−0.090 *(0.046)
Region	0.089 (0.129)	0.081 (0.129)	0.612 **(0.296)	0.173 (0.134)
Gender	0.249 ***(0.086)	0.241 ***(0.086)	0.210 (0.155)	0.243 ***(0.089)
Migration	−0.126 (0.106)	−0.130 (0.106)	−0.386 **(0.172)	−0.038 (0.109)
Economic status	0.133 (0.130)	0.127 (0.130)	−0.232 (0.172)	−0.089 (0.142)
Elderly support	0.031 (0.084)	0.027 (0.084)	−0.060 (0.192)	−0.040 (0.129)
Constant	0.369 (0.362)	0.479 (0.362)	0.454 (0.396)	0.409 (0.396)
n	2039	2039	2039	2039
Pseudo R^2^	0.175	0.177	0.247	0.097

*p < 0.1; **p < 0.05; ***p < 0.01, the following table is the same.

To assess robustness, Models M3 and M4 use binary indicators for first-birth and second-birth intentions, respectively. Following the CGSS 2023 item “If there were no policy restrictions, how many children would you want?,” the First-Birth Intention equals 1 if the respondent wants at least one child (0 otherwise), and the Second-Birth Intention equals 1 if the respondent wants at least two children (0 otherwise). In Model M3, the coefficient on Physical Exercise is 0.153 (SE = 0.074), positive and significant at the 5% level. In Model M4, the coefficient is 0.070 (SE = 0.039), positive and significant at the 10% level. These results collectively support hypothesis 1.

#### 4.2.2 Path analysis

##### 4.2.2.1 Health effect

Table 4 reports the estimates results. In Models M11–M13, the coefficient on Physical Exercise is positive and statistically significant for all three health outcomes—Overall health (*β* = 0.118, *p <* 0.05), Physical Health (*β =* 0.091, *p* < 0.10), and Mental Health (*β* = 0.112, *p <* 0.05)—indicating that greater participation is associated with better health.

**Table 4:** Results of the test for health effect mechanism.

Variables	Overall health	Physical health	Mental health	Fertility attitude			
	M11	M12	M13	M14	M15	M16	M17
Physical exercise	0.118 ** (0.051)	0.091 * (0.053)	0.112 ** (0.050)	0.079 * (0.036)	0.074 * (0.038)	0.071 ** (0.034)	0.070 ** (0.036)
Overall health level				0.045 * (0.024)			0.044 * (0.025)
Physical health level					0.012 (0.013)		0.010 (0.012)
Mental health level						−0.124 (0.091)	−0.120 (0.090)
Control variables	Yes	Yes	Yes	Yes	Yes	Yes	Yes
Constant	−4.980 *** (0.540)	−4.632 *** (0.587)	−4.412 *** (0.559)	−0.316 (0.377)	−0.322 (0.374)	−0.223 (0.373)	−0.457 (0.416)
n	2039	2039	2039	2039	2039	2039	2039
Pseudo R^2^	0.052	0.031	0.017	0.179	0.171	0.173	0.180

In the mediation models for Fertility Attitude (M14-M17), Physical Exercise remains positive and significant (M14: *β* = 0.079, *p <* 0.10; M15: *β* = 0.074, *p* < 0.10; M16: *β* = 0.071, *p <* 0.05; M17: *β =* 0.070, *p <* 0.05). Among the mediators, Overall Health is positively associated with Fertility Attitude (M14: *β* = 0.045, *p* < 0.10; M17: *β =* 0.044, *p* < 0.10), whereas Physical Health (M15: *β* = 0.012; M17: *β* = 0.010) and Mental Health (M16: *β* = −0.124; M17: *β* = −0.120) are not statistically significant.

Taken together, these findings are consistent with a partial mediation mechanism: physical exercise improves health, and overall health in turn raises Fertility Attitude, thereby supporting hypothesis 2.

##### 4.2.2.2 Harmony effect

Table 5 shows that physical exercise is positively associated with both outcomes: in M18 (Trust) *β* = 0.095, *p* < 0.10; in M19 (Happiness) *β* = 0.091, *p* < 0.10.

**Table 5:** Results of the test for harmonious effect mechanism.

Variables	Trust	Happiness	Fertility attitude		
	M18	M19	M20	M21	M22
Physical exercise	0.095 * (0.052)	0.091 * (0.053)	0.074 ** (0.036)	0.076 ** (0.036)	0.079 ** (0.036)
Trust			0.124 ** (0.042)		
Happiness			0.001 (0.064)	0.128 *** (0.043)	
Control variables	Yes	Yes	Yes	Yes	Yes
Constant	−2.812 *** (0.480)	−6.230 *** (0.596)	−0.628 (0.396)	−0.274 (0.429)	−0.504 (0.435)
n	2039	2039	2039	2039	2039
Pseudo R^2^	0.006	0.023	0.182	0.177	0.181

In the model, adding the mediators slightly attenuates the sports coefficient relative to the baseline without mediators (M22: *β* = 0.079, *p* < 0.05): with both mediators (M20) *β =* 0.074, *p <* 0.05; with Happiness only (M21) *β* = 0.076, *p* < 0.05. Among the mediators, Trust is positive and significant when included with happiness (M20: *β* = 0.124, *p <* 0.05), whereas Happiness is positive and significant when entered alone (M21: *β* = 0.128, *p <* 0.01). These patterns indicate partial mediation, supporting hypothesis 3.

**Figure 1:**
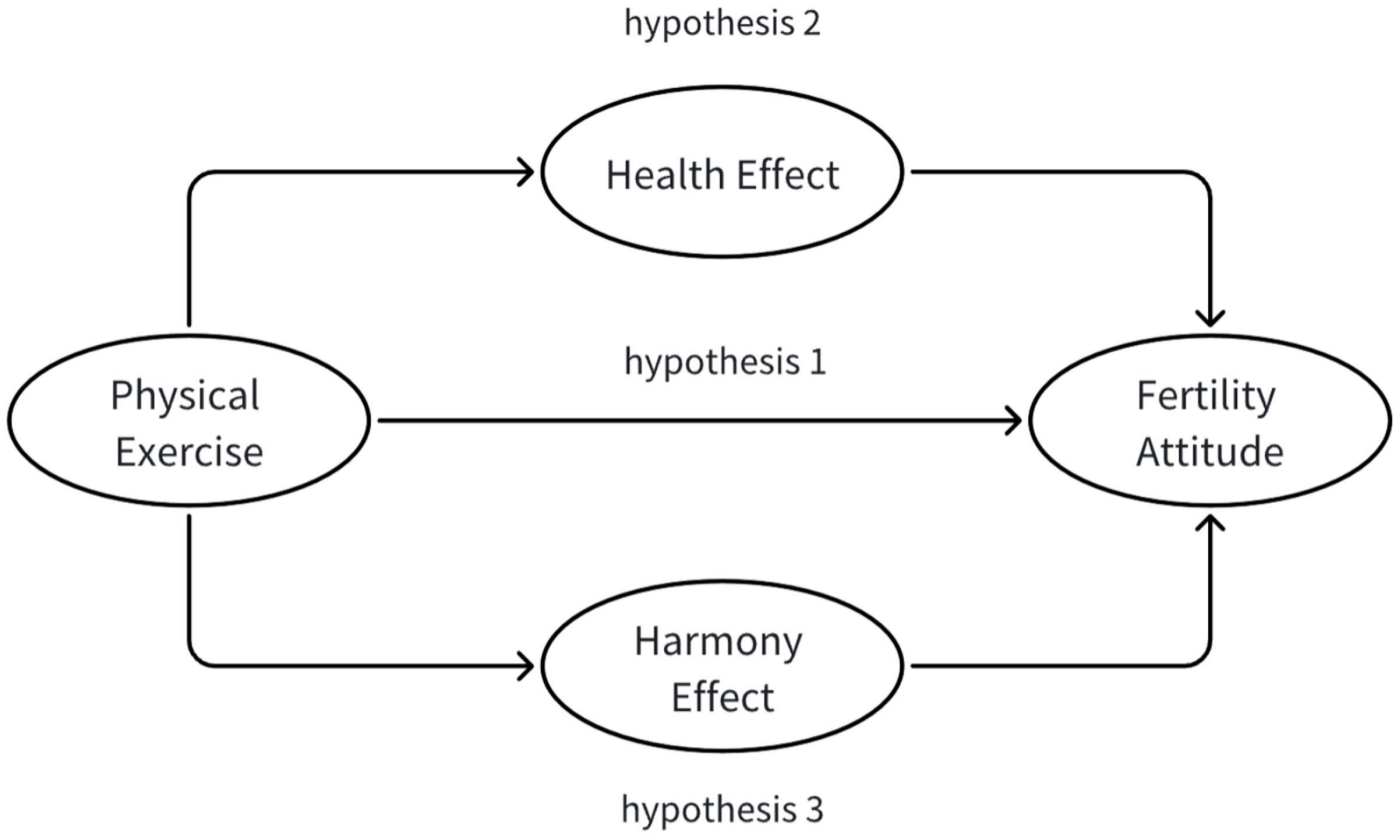
Conceptual framework: pathways from physical exercise to fertility attitudes.

Building on the foregoing results, we delineate a conceptual framework that maps the pathways from physical exercise to fertility attitudes (Figure 1).

#### 4.2.3 Moderation analysis

Discussion of hypothesis 4. Physical exercise require both economic and temporal resources, so financial and time constraints may confound the link between physical exercise and fertility attitude. In the affluence split, the coefficient on Physical Exercise is 0.054 (not significant) in the affluent subsample (M5) and 0.118 (*p* < 0.10) in the non-affluent subsample (M6), indicating a modest positive association among resource-constrained households. In the overtime split, the coefficient is 0.141 (*p* < 0.10) for overtime workers (M7) and −0.058 (not significant) for those not working overtime (M8), suggesting that the association appears among individuals who maintain exercise despite time pressure, consistent with a selection or health-orientation channel rather than simple time abundance.

In the money-time status split, the coefficient is 0.345 (*p* < 0.05) for the money-rich group (M9) and 0.172 (not significant) for the time-rich group (M10), implying that financial capacity strengthens the relationship whereas surplus time alone does not. Overall, hypothesis 4 is not supported in its strict cost-compression form: physical exercise is positively related to fertility attitudes, but the effect depends on the resource mix-stronger with financial capacity and among those who persist in exercising under time pressure (Table 6).

**Table 6:** The influence of time and economic factors on the relationship between physical exercise and fertility attitude.

Variables	Fertility attitude					
	Affluent vs. non-affluent		Works overtime vs. non-works overtime		Money-rich vs. time-rich	
	M5	M6	M7	M8	M9	M10
Physical exercise	0.054 (0.047)	0.118 * (0.060)	0.141 * (0.081)	−0.058 (0.097)	0.345 ** (0.130)	0.172 (0.184)
Control variables	Yes	Yes	Yes	Yes	Yes	Yes
Constant	−0.006 (0.545)	−0.346 (0.671)	−1.705 (1.025)	0.402 (1.026)	−1.648 (1.665)	−0.476 (1.658)
n	1,087	957	275	259	150	133
Pseudo R^2^	0.174	0.168	0.187	0.259	0.235	0.284

### 4.3 Robustness test

#### 4.3.1 Work–life conflict as a moderator

To examine whether work–life conflict moderates the association between physical exercise and fertility attitudes, we construct a binary indicator from two items—“Your work interferes with your family life” and “Your family life interferes with your work.” Responses of “rarely/never” are coded 0 (no conflict), and all other responses are coded 1 (conflict). Subsample regressions are reported in Table 7. In the no-conflict subsample (M23), the coefficient on physical exercise is 0.060 (n.s.); in the conflict subsample (M24), the coefficient is 0.113 (*p* < 0.10), indicating a positive association when work–life conflict is present. The result is robust across alternative specifications: M25 = 0.035 (*p* < 0.05), M26 = 0.088 (*p* < 0.05), M27 = 0.069 (*p* < 0.10), M28 = −0.508 (*p* < 0.10), M29 = 0.064 (*p* < 0.10), M30 = 0.198 (*p <* 0.05), and M31 = 0.188 (*p <* 0.05). These estimates consistently support hypothesis 1.

**Table 7:** Robustness analysis.

Variables	Z1				Z2	Z3	Z1	Z4	
	M23	M24	M25	M26	M27	M28	M29	M30	M31
H₁	0.060 (0.048)	0.113 * (0.091)	0.035 ** (0.016)	0.088 ** (0.041)	0.069 * (0.037)	−0.508 * (0.273)		0.198 ** (0.095)	
H₂						0.185 * (0.099)			
H₃							0.064 * (0.033)		0.188 ** (0.094)
Control variables	Yes	Yes	/	/	Yes	Yes	Yes	Yes	Yes
Constant	−0.182 (0.488)	−0.332 (0.712)	−0.259** (0.107)	0.429* (0.221)	−2.546** (0.569)	−0.247 (0.352)	−0.329 (1.645)	−0.505 (1.687)	−0.512 (1.690)
*n*	1,145	894	2039	2,039	1,132	1,132	2,039	554	454
Pseudo R^2^	0.174	0.049	0.042	0.159	0.150	0.151	0.129	0.119	0.121

#### 4.3.2 Endogeneity discussion

We acknowledge that, even with extensive individual-, household-, and society-level controls, the baseline models may still suffer from endogeneity. Two main sources are: (1) reverse causality—physical exercise may affect re-fertility attitudes, but stronger fertility attitudes may also encourage physical exercise; and (2) omitted variables—unobserved traits (e.g., personality or emotional disposition) could jointly influence both physical exercise and fertility attitudes.

To address these concerns, we implement an instrumental-variables (IV) strategy using four instruments: (i) household car ownership; (ii) number of air trips in the past 12 months (round-trip counted once); (iii) weekly private motor-vehicle usage (hours; excluding public transport); and (iv) the mean sports-participation frequency of other respondents in the same city (excluding the individual). These instruments satisfy relevance because private mobility and a sports-active social milieu plausibly raise participation; exogeneity is supported by the lack of a direct causal link from fertility attitudes to these instruments.

We estimate IV regressions via two-stage least squares (2SLS) and an IV-probit model; results appear in Table 7 as M25 (2SLS) and M26 (IV-probit). In both specifications, the coefficient on physical exercise remains positive and statistically significant, reaffirming hypothesis 2. Diagnostic tests support instrument validity: Shea’s partial *R*^2^ = 65.65% and the first-stage *F* = 596.76 reject weak instruments; the overidentification test yields *p* = 0.2966, consistent with joint exogeneity. These results indicate that the IV strategy effectively mitigates endogeneity and that the positive effect of physical exercise on fertility attitudes is robust.

#### 4.3.3 Sample selection and variable sensitivity analysis

We refine the robustness checks and relabel models to match Table 7 (M23–M31). First, under alternative binary codings of the dependent variable Z1 (M23–M25), the coefficient on physical exercise is 0.060 (n.s.) in M23, but remains positive and significant in M24 (*β* = 0.113, *p* < 0.10) and M25 (*β* = 0.035, *p* < 0.05). Second, when excluding respondents with zero fertility attitudes to construct Z2, results are positive: M26 reports *β*(H1) = 0.088 (*p* < 0.05), and M27 reports *β*(H1) = 0.069 (*p* < 0.10) with the squared term also significant *β*(H2) = 0.185 (*p* < 0.10). Third, a stricter threshold specification (Z3) yields M28 with *β*(H1) = −0.508 (*p* < 0.10).

Next, replacing frequency with preference (H3) shows a positive association: M29 (with Z1) reports *β*(H3) = 0.064 (*p* < 0.10). Using the ordinal outcome Z4, the coefficients remain positive and significant: M30 (frequency, H1) *β* = 0.198 (*p* < 0.05) and M31 (preference, H3) *β* = 0.188 (*p* < 0.05). Overall, across the majority of specifications (M2-M27, M29-M31), physical exercise is positively and significantly related to fertility attitudes, supporting the robustness of hypothesis 1.

## 5 Conclusion, limitations

### 5.1 Conclusion

Our evidence shows that physical exercise is positively associated with fertility attitudes among individuals of reproductive age, and that this relationship is transmitted through two empirically supported pathways: a health pathway and a harmony pathway. Importantly, the association is not a simple “cost-compression” effect of time and money; rather, it is contingent on the resource mix, appearing stronger when financial capacity is present and among individuals who continue to exercise despite time pressure, but not among those who are merely time-rich. Taken together, these findings imply that exercise likely improves fertility attitudes by augmenting physical and socio-emotional resources, and that the strength of this mechanism depends on whether economic constraints are relaxed and whether individuals can sustain activity under binding schedules.

Translating these results to the individual level, the salient design principle is to privilege frequency and sustainability over volume. Moderate-intensity routines that are feasible under ordinary time pressure—approximately 20–30 min three times per week, or 10–15 min micro-sessions anchored to commuting or lunch breaks—are most likely to accumulate health gains while remaining adherent. To activate the harmony pathway, exercise should be socially embedded: stable partners, small fixed groups, or recurring community classes reliably enhance interpersonal contact and subjective well-being. Because the association strengthens when financial frictions are low or when persistence under time pressure is demonstrated, individuals should minimize logistical costs by choosing proximal or home-based options and—where feasible—make small, earmarked financial commitments (e.g., low-cost class packs or basic equipment) that increase follow-through; integrating childcare or household participation reduces schedule conflicts and further supports adherence. By contrast, expanding unstructured leisure time without reducing monetary/logistical frictions or adding social structure is unlikely to reproduce the same gains.

Implementation can be specified as a brief, mechanism-aligned protocol. Individuals establish a fixed weekly schedule that survives routine workload variability (e.g., two short weekday sessions linked to commute or lunch and one longer session on the weekend), select nearby venues to cap access time, and pair sessions with a consistent partner or small group to create social accountability. Budget-constrained contexts can rely on outdoor or body-weight programs while preserving the same scheduling regularity and social embedding; financially unconstrained contexts may add minimal equipment or prepaid sessions that increase commitment without materially altering intensity. To verify that the intended mechanisms are engaged, individuals track simple pre–post indicators aligned with the mediators—self-rated health, recent activity limitation, low-mood frequency, interpersonal trust, and life satisfaction—at two- to four-week intervals alongside adherence records. Mechanism-consistent improvements on these scales, rather than duration alone, should guide subsequent adjustment of frequency or social format.

Several caveats qualify these recommendations. The underlying estimates are cross-sectional and based on self-reports, so the guidance is correlational and should be tailored to baseline health status; individuals with relevant medical conditions should seek clinical clearance and progress gradually. Cultural and contextual factors may shape how easily the harmony pathway is activated and which social formats are sustainable. Within these constraints, prioritizing habit stability, proximity, low friction, and structured social engagement offers the clearest individual-level route to strengthen the health and harmony mechanisms through which exercise relates to more positive fertility attitudes.

### 5.2 Limitation

First, although the study emphasizes fertility attitudes as a key variable in predicting fertility behavior, it has not fully bridged the gap between fertility attitudes and actual fertility behavior. Future research will explore how to more effectively convert fertility attitudes into specific reproductive actions by leveraging the positive effects of physical activity.

Second, the cross-sectional data used in this study can only reveal the immediate relationship between physical exercise and fertility attitudes, and cannot track the long-term impact of participation in physical activities on changes in fertility attitudes over time. Future studies should adopt longitudinal designs or experimental methods to explore the dynamics of causality.

Third, given the strong correlation between personality traits and psychological and behavioral changes, this study lacks data on individual personality characteristics. Future research will consider incorporating these variables for a more comprehensive analysis to better understand how physical activity influences fertility attitudes and behavior by affecting individuals’ psychological and behavioral traits.

Fourth, since the dataset does not capture the relationship between spouses, the data on family happiness and marital satisfaction used in this study are based on ratings from one or both spouses, which may carry subjective bias. Future studies will employ more precise quantitative methods and integrate both primary and secondary data to comprehensively assess the actual experiences of family members.

## Data Availability

The datasets presented in this study can be found in online repositories. The names of the repository/repositories and accession number(s) can be found at: http://www.cnsda.org/index.php?r=projects/view&id=65635422.
